# A Rare Case of Nipple–Areolar Complex Partial Necrosis following Micropigmentation: What to Learn?

**DOI:** 10.1097/GOX.0000000000002494

**Published:** 2019-11-27

**Authors:** Marta Starnoni, Massimo Pinelli, Gianluca Franceschini, Giorgio De Santis

**Affiliations:** From the * Division of Plastic Surgery, University of Modena and Reggio Emilia, Modena, Italy; † Division of Breast Surgery, Catholic University, Rome, Italy.

## Abstract

A 52-year-old woman, without any comorbidity, presented at our institution for reconstruction of nipple–areolar complex (NAC). Nipple reconstruction was obtained through local skin flaps. After 2 months, a tattoo of the NAC was performed. Follow-up was planned at 6 months. Nevertheless, the patient came to our attention 2 days after tattooing for partial necrosis of the epidermal–dermal layer of the tattooed area with partial muscular layer exposition. Empirical antibiotic treatment was immediately started to avoid infection. Daily medications were performed for 3 weeks. Complete healing was obtained within 3 weeks without the necessity of a skin graft. We think that the partial necrosis of the NAC occured because of vascular impairment of the dermal and subdermal vascular plexus during micropigmentation. From this experience, we developed some advice to improve our clinical practice by allowing surgeons, especially if trainees, to avoid complications in performing NAC micropigmentation.

Nipple–areolar complex (NAC) reconstruction and dermal tattooing are usually performed in addition to breast reconstruction with the aim to restore the patient's body image. These final steps of breast reconstruction are fundamental to achieve a natural, esthetic, and symmetric result.^1,2^

Micropigmentation is a relatively easy procedure that provides permanent camouflage, and it is considered to be devoid of any significant adverse effects. The pigments used in the process of micropigmentation are usually inert, nontoxic, nonallergenic, and tissue stable.^3^

Even if micropigmentation is considered a safe procedure, it cannot be considered free of potential severe complications, which should be promptly recognized, individualized, and treated. We report a rare case of a skin-necrotizing reaction after NAC micropigmentation. To our knowledge, similar cases have not been previously reported.

## CASE

**Fig. 1.**
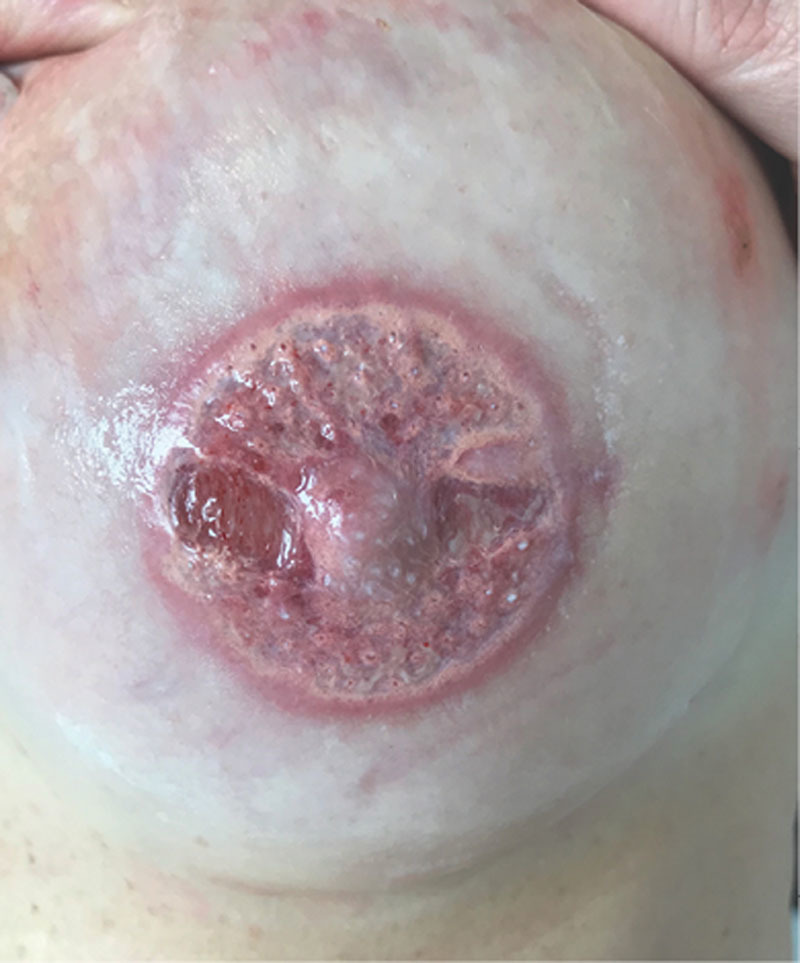
Subtotal necrosis of dermis with exposition of the muscular layer after NAC micropigmentation. Residual dry scar ointment seems to be as micro-pustules.

**Fig. 2.**
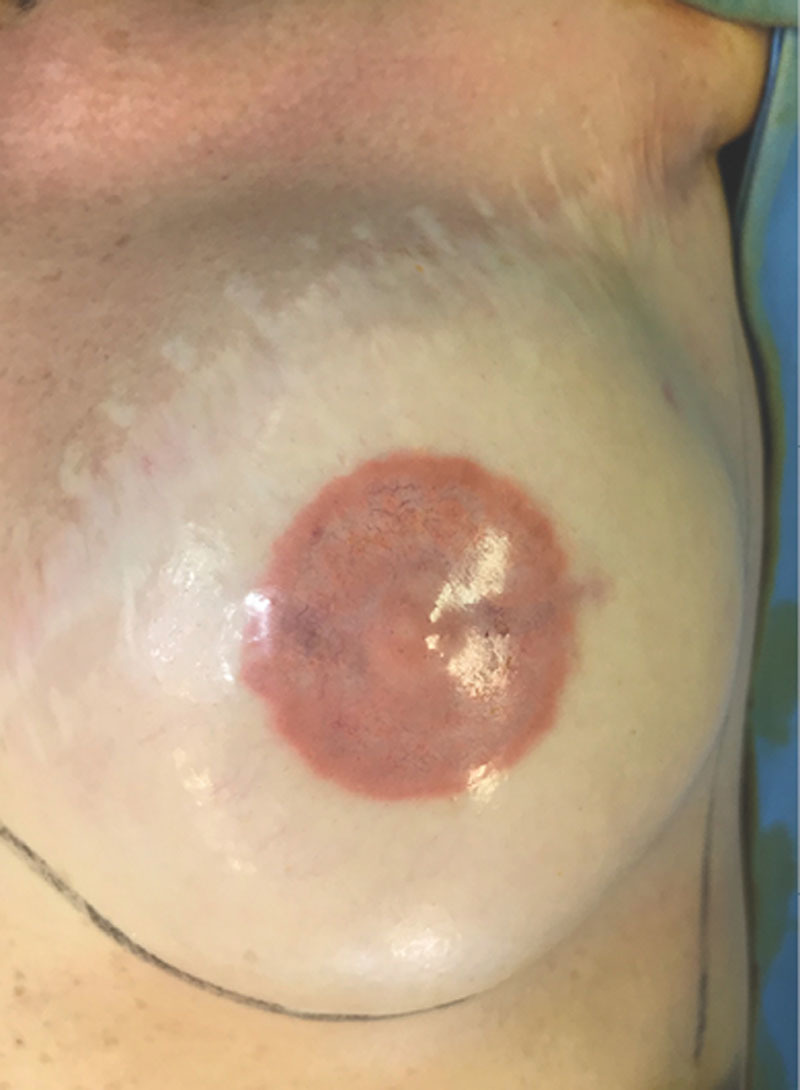
Complete healing after 3 weeks of daily medications.

A 52-year-old woman, without any comorbidity, presented at our institution for reconstruction of NAC. A left breast mastectomy, followed by breast implant reconstruction, was performed 15 years before without radiation therapy. Nipple reconstruction was obtained through local skin flaps. After 2 months, a tattoo of the NAC was performed under local anesthesia (2% lidocaine with epinephrine) using a micropigmentation machine (Amiea, Berlin, Germany), and pigment colors were made by the same manufacturers. Before tattooing, the patient did not report any constitutional symptoms, she had no known drug allergies, she had had no previous tattoos, and she underwent previous minor procedures under local anesthesia. During the procedure, we observed appropriate skin antisepsis, we used a sterile disposable needle and single-use colors and maintained strict disinfection of equipment and surfaces. Adrenaline is usually used because it limits the bleeding that blurs distinctions between the colors of the pigments. After tattooing, the patient was advised to perform daily medication with boric water solution for 5 days and then daily scar ointment application for 2 months. Follow-up was planned at 6 months. Nevertheless, the patient came to our attention 2 days after tattooing for pain and pigment secretion. Clinical assessment revealed partial necrosis of the epidermal–dermal layer of the whole tattooed area with full-thickness skin loss in correspondence with the nipple reconstruction donor site scar, where muscle layer exposure could be noted (Fig. 1). She did not refer pruritus of the tattooed area. Empirical antibiotic treatment (amoxicillin–clavulanic acid 1 g, twice for 6 days) was immediately started to avoid infection. Daily medication with topic antibiotic ointment and boric water solution was performed for 1 week. For the following 2 weeks, the patient was advised to daily apply a physiologic solution wet dressing. Complete healing with hyperpigmentation and little reduction in nipple projection (Fig. 2) was obtained within 3 weeks without the necessity of a skin graft.

## DISCUSSION

Due to the lack of any risk factors for possible difficult healing (such as previous radiotherapy, diabetes, obesity, smoking), the surgeon did not give proper attention to the extreme thinness of the skin, despite extensive tattooing experience and training.

We think that the partial necrosis of the NAC occured because of vascular impairment of the dermal and subdermal vascular plexus during micropigmentation. Furthermore, the vascularization was not totally restored after surgical nipple reconstruction performed 2 months before.

Dermal and subcutaneous layers were extremely thin. Micropigmentation was performed by adjusting an excessive length of needle with subsequent vascular impairment. In fact, regulation of needle length varies from patient to patient according to surgeon's evaluation of the dermal layer thickness.

After the necrosis occurrence, the patient referred that she suffered from an important intolerance to medical tape limited to the reconstructed breast. Nevertheless, the clinical findings were not suggestive of allergenic reactions. In fact, allergic reaction (including pruritus, burning sensation, itching, stinging, erythema, edema) has not been observed. Moreover, our patient had no previous history of sensitization to pigments. Local anesthesia allergy can be excluded because of the previous minor procedures she had.

The hypothesis of a primitive infection that had secondarily led to necrosis was refused because of the short time to onset and the lack of infection signs. Infectious complications occur within 4–22 days and range from mild cellulitis and small pustules to abscesses.^4,5^

Very few examples of cutaneous necrotizing secondary to a permanent decorative tattoo have been found in the literature.^6^ At the complete healing, we noted a hyperpigmentation of the NAC of our patient. A case of ulceration of the skin at the site of permanent black tattoo on forearm and subsequent replacement of the pigment with hyperpigmented healthy skin has been previously reported.^7^ Hyperpigmentation is a sign of scar tissue neo-angiogenesis.

Radiotherapy can be considered an important risk factor for skin slough but this patient did not receive radiation therapy.

From this experience, we developed some advice to improve our clinical practice by allowing surgeons, especially if trainees, to avoid complications in performing NAC micropigmentation:

- During the nipple reconstruction through skin flaps, it is important to record whether a particular thin cutaneous layer is noted, so that during the future tattooing a short needle length can be set.
- In these selected patients, it is important to wait 6–9 months after nipple reconstruction before tatooing: a total restoration of the vascularization of the skin flaps is necessary, and a total scar stabilization is recommended to avoid dehiscence after tattooing.
- Ask the patient for an eventual intolerance to medical tape of the reconstructed breast that can be a symptom of sensitive skin.
- Do not use epinephrine during local infiltration of anesthesia so that if you note significant bleeding during the micropigmentation, a vascular impairment could be suspected. Subsequently, the procedure should be stopped and repeated at 6-month follow-up, if necessary.
- In suspected cases, a 3-day follow-up after tattooing is recommended: a timely treatment of partial necrosis (with systemic and topic antibiotics and daily medications) allows complete healing without the risk of infection.

Furthermore, we think that micropigmentation should be performed by well-qualified health-care workers under the supervision of a physician. In fact, breast skin after reconstruction has to be considered delicate and sensitive because of previous mastectomy with subsequent scar tissue formation and, in many cases, for the previous radiotherapy, for thin skin flaps and for the presence of a breast implant. Even if medical staff did not have professional tattooer skills, the presence of a physician is important to take appropriate measures in selected patients and to promptly recognize, individualize, and treat potential complications. It should be added that, in Italy, nipple–areola tattoo is a reimbursed procedure, and it can be performed only by health-care workers in hospital or in any other health-care facility.

## CONCLUSIONS

Micropigmentation is considered to be an easy and safe technique. Nevertheless, even if the patient has no risk factors such as diabetes, obesity, and heavy smoking that alert the surgeon to potential complications, particular attention must be given to local conditions of skin atrophy and recent cicatrization. In fact, in these cases, further advice to routine clinical practice must be considered to reduce the risk of NAC necrosis following micropigmentation.
